# Using a laryngoscope and endotracheal tube succeeds in a difficult case of nasogastric tube insertion

**DOI:** 10.1186/cc14284

**Published:** 2015-03-16

**Authors:** J Park, Y Lee

**Affiliations:** 1 Ewha Womans Unversity Hospital, Seoul, South Korea

## Introduction

Nasogastric (NG) tube insertion is necessary in a variety of critically ill patients for intra-abdominal decompression, prevention of aspiration, route of medication administration and nutrition. However, it often fails in patients who showed sedated or comatose mentality with poor cooperation during the procedure. Although there are many reports inserting a NG tube in difficult cases, most methods need a special guide wire, tube or nasoendoscope. We report a case of NG tube insertion in a comatose patient using a laryngoscope and endotracheal tube which are easily available.

## Methods

A NG tube was inserted using a laryngoscope and endotracheal tube.

## Results

**Figure 1:**
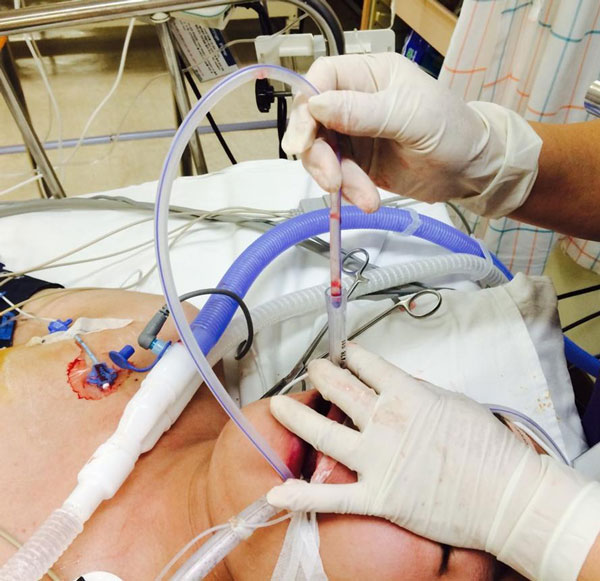
**Insertion of a nasogastric tube through an endotracheal tube in the esophagus**.

A 15-year-old male patient admitted due to abrupt mental change and brain imaging showed severe subdural hemorrhage. NG tube insertion was done for enteral feeding but failed several times though changing position. As we had no guide wire and no nasoendoscope, an endotracheal tube was used as guidance for the NG tube. After making a longitudinal midline cut on the endotracheal tube, it was inserted into the esophagus under a laryngoscope. The NG tube was pushed into the endotracheal tube, and then the endotracheal tube was removed through the cut, reserving the NG tube. We checked the position of the NG tube by air sound and X-ray, and started enteral feeding without complication, such as nasal bleeding, emphysema, and gastric perforation. See Figure 1.

## Conclusion

We report a new method of NG tube insertion using a laryngoscope and endotracheal tube.

